# p16, pRb, and p53 in Feline Oral Squamous Cell Carcinoma

**DOI:** 10.3390/vetsci3030018

**Published:** 2016-08-18

**Authors:** Wachiraphan Supsavhad, Wessel P. Dirksen, Blake E. Hildreth, Thomas J. Rosol

**Affiliations:** Department of Veterinary Biosciences, College of Veterinary Medicine, The Ohio State University, 1925 Coffey Road, Columbus, OH 43210, USA; supsavhad.1@osu.edu (W.S.); dirksen.8@osu.edu (W.P.D.); behildrethiii@aol.com (B.E.H.)

**Keywords:** feline, oral, squamous cell carcinoma, p16, p53, pRb, immunohistochemistry

## Abstract

Feline oral squamous cell carcinoma (FOSCC) is a highly aggressive head and neck cancer in cats, but the molecular pathogenesis of this cancer is still uncertain. In this study, p16, p53, and pRb proteins were detected and quantified by immunohistochemistry in forty-three FOSCC primary tumors and three FOSCC xenografts. p16 mRNA levels were also measured in three FOSCC cell lines (SCCF1, F2, and F3), which were consistent with their p16 immunoreactivity. Feline SCCF1 cells had very high levels of p16 protein and mRNA (55-fold greater) compared to SCCF2 and F3. A partial feline p16 cDNA sequence was amplified and sequenced. The average age of cats with FOSCC with high p16 immunoreactivity was significantly lower than the average age in the low p16 group. Eighteen of 43 (42%) FOSCCs had low p16 intensity, while 6/43 (14%) had high p16 immunoreactivity. Feline papillomavirus L1 (major capsid) DNA was not detected in the SCC cell lines or the FOSCCs with high p16 immunostaining. Five of 6 (83%) of the high p16 FOSCC had low p53, but only 1/6 (17%) had low pRb immunoreactivity. In summary, the staining pattern of p16, p53, and pRb in FOSCC was different from human head and neck squamous cell carcinoma and feline cutaneous squamous cell carcinoma. The majority of FOSCCs have low p16 immunostaining intensity, therefore, inactivation of CDKN2A is suspected to play a role in the pathogenesis of FOSCC. A subset of FOSCCs had increased p16 protein, which supports an alternate pathogenesis of cancer in these cats.

## 1. Introduction

Feline oral squamous cell carcinoma (FOSCC) is one of the most common cancers in cats [1]. Over 70% of FOSCC invade the adjacent mandible or maxilla, resulting in bone lysis and a short survival time (less than one year) [2] (pp. 302–312). Pathogenesis of this cancer is not well-understood and few treatment options are available with limited success.

FOSCC and human head and neck cancer (HNSCC) have a high degree of biological similarity including location of tumors and bone-invasive behavior [3] (pp. 7–16), [4] (pp. 125–136), [5] (p. 218). In humans, HNSCC can be classified in two major categories; human papillomavirus (HPV)-induced and non-viral-associated HNSCC [6] (pp. 9–22). A favorable prognosis as well as good treatment response was commonly reported in patients with the HPV HNSCC, while most patients with non-viral-associated HNSCC have a poor prognosis. Inactivation or mutation of tumor suppressor genes, especially CDKN2A and TP53, were reported in more than 60% of non-viral associated HNSCC patients [6] (pp. 9–22).

TP53 is a tumor suppressor gene that encodes p53 protein and has an important role in regulating cell cycle and tumor progression. More than 50% of HNSCC patients were reported to have either TP53 mutations or increased p53 protein (usually due to mutations) detected by immunohistochemistry, which were associated with a poor clinical outcome and opposing tumor progression [6] (pp. 9–22), [7] (pp. 73–122). High p53 immunostaining was reported in 43% (10/23) of FOSCCs, while 35% (8/23) of FOSCCs did not have high p53 staining [8] (pp. 209–214).

Low p16 protein immunoreactivity was found in 48%–83% of human HNSCCs [9] (pp. 58–60), [10] (pp. 1931–1934), [11] (pp. 3630–3633), while high p16 immunoreactivity was found in 37%–59% of human HNSCC [10] (pp. 1931–1934), [12] (pp. 649–655). Loss of p16 protein immunoreactivity in human HNSCC correlated with CDKN2A inactivation, especially point mutations, promoter hypermethylation, or homozygous deletion [6] (pp. 9–22), [11] (pp. 3630–3633). In contrast, increased p16 immunoreactivity correlated with high-risk-human papilloma virus (HPV)-induced HNSCC [13] (pp. 2166–2173).

Papillomavirus (PV) is a small, double-stranded circular DNA virus. Its genome has non-coding upstream, early, and late regions. The early region consists of 8 genes (E1 to E8). In high-risk human PVs (HPV), including HPV-16, -18 and -31, E6 and E7 are oncogenic and cause degradation of p53 and pRb during the cell cycle [14] (pp. 2). An association between the presence of feline catus papillomavirus type 2 (FcaPV2) DNA and p16 and pRb immunostaining in preneoplastic and neoplastic cutaneous SCC lesions has been reported in cats [15] (pp. 538–545). Increased p16 with decreased pRb was suggested to be a marker for FcaPV in feline cutaneous SCC [15] (pp. 538–545). However, the presence of papillomavirus and the relationship between p16, p53, and pRb and papillomavirus infection in FOSCC are still unclear. Moreover, FOSCC subtypes have not been classified in this species. We hypothesized that FOSCC could be classified in a manner similar to HNSCC using the immunohistochemical (IHC) pattern and intensity of p16, pRb, and p53 staining. High p16 with a low pRb and p53 immunostaining profile was expected in PV-related FOSCC, while low p16 and either a high or low p53 IHC pattern were proposed in non-PV FOSCC. The goals of our study were to investigate the (1) pattern of immunoreactivity to p16, pRb, and p53 in FOSCC; (2) determine the presence of papillomavirus DNA in 3 FOSCC cell lines and formalin-fixed, paraffin-embedded (FFPE) FOSCC samples with high p16 immunostaining intensity; (3) correlate p16 immunoreactivity and mRNA expression in FOSCC cell lines; and (4) sequence a region of feline p16 cDNA.

## 2. Materials and Methods

### 2.1. Sample Collection

Forty-three FFPE FOSCC specimens originating from the gingiva (*n* = 9), tongue (*n* = 11), sublingual region (*n* = 12), and mandible or maxilla (*n* = 11) were obtained. Three FOSCC cell lines (SCCF1, SCCF2 and SCCF3) previously developed by our laboratory [16] (pp. 676–683), [17] (pp. 491–499) and xenograft tissues from nude mice with SCCF1, 2, and 3 were also used. Since positive p16 immunostaining and PV DNA has been reported in feline Bowenoid in situ carcinoma (FBISC) [15] (pp. 538–545), a FBISC was used as a positive control for p16 and PV DNA. One normal cat gingiva was used for the qRT-PCR, and two normal cat gingiva FFPE samples were used to compare to the FOSCC IHC results.

### 2.2. Immunostaining

Forty-three FOSCC FFPE, 3 FOSCC xenografts, 1 FBISC and 2 cat gingival tissues were cut into 5-µm sections, deparaffinized, rehydrated, and incubated in antigen retrieval solution (Dako, Carpinteria, CA, USA) at 80 °C for 40 min. and cooled to room temperature for 10 min. Hydrogen peroxide (3%) was used to block endogenous peroxidase and serum-free protein block (Dako) was applied. Samples were incubated overnight at 4 °C with either (1) mouse anti-human p16 monoclonal antibody [18] (pp. 280–283) (BD Bioscience, clone G175–405, catalog number: 550834; 1:30) in phosphate-buffered saline (PBS); (2) mouse anti-human non-phosphorylated retinoblastoma (pRb) antibody [15] (pp. 538–545) (BD Bioscience, clone G3–245, catalog number: 554136; 1:40) in PBS; or (3) mouse anti-human p53 antibody [15] (pp. 538–545) (BD Bioscience, clone PAb 240, catalog number: 554166; 1:100) in PBS. The anti-pRb antibody was specific for nuclear pRb. Anti-mouse monoclonal secondary antibody (Vector Laboratories, catalog number: BA-9200) was used at 1:200 in PBS for 30 min. Slides were stained with Vector ABC Elite complex (Dako) for 30 min, diaminobenzidine tetrahydrochloride (Dako) for 5 min, and with Mayer’s hematoxylin for 1 min.

The highest intensity pRb and p53 immunostained samples were used as positive controls, while omission of the primary antibody was used as a negative control. Normal tissue surrounding each tumor served as an internal control. IHC staining of p16, pRb, and p53 was classified into one of 3 categories: (1) absent to low; (2) moderate; and (3) high immunoreactivity, according to the average percentage of immunostained cells and the intensity level of the immunostaining in 5 different 400× fields (Table 1).

### 2.3. p16 mRNA in 3 FOSCC Cell Lines

RNA was purified from SCCF1, SCCF2, and SCCF3 cells and a normal cat gingiva using the Absolutely RNA RT-PCR miniprep kit (Agilent Technologies, Santa Clara, CA, USA). Normal cat gingiva was collected from a cat euthanized for other reasons, snap frozen in liquid nitrogen, and stored at −80 °C.

**Figure 1:**
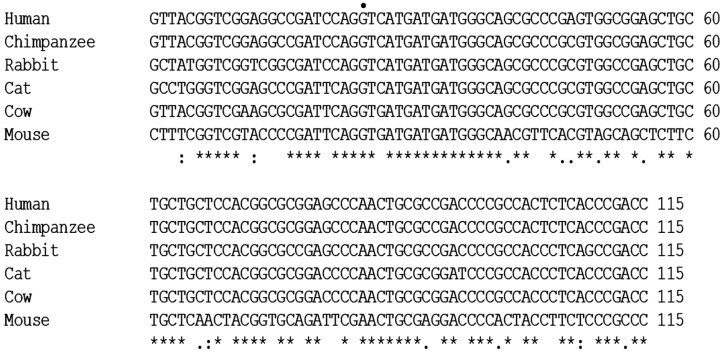
Alignment between human, chimpanzee, rabbit, cat, cow and mouse p16 cDNA sequences. The 115-nucleotide RT-PCR amplicon spanning the junction between exons 1α and 2 of the cat p16 cDNA was aligned with the corresponding regions of p16 from the indicated species. The dot indicates the first nucleotide of exon 2. An asterisk (*) indicates a nucleotide that is identical between all the aligned sequences, a colon (:) indicates a nucleotide of strongly similar properties between aligned sequences and a period (.) indicates a nucleotide of weakly similar properties between aligned sequences. Alignment was performed using ClustalW2 software (European Bioinformatics Institute ftp server).

The quantitative RT-PCR (qRT-PCR) conditions have previously been described [19] (pp. 1251–1265). A consensus p16 cDNA sequence (not shown) of exons 1α (126 nucleotides) and 2 (307 nucleotides) was obtained by aligning p16 cDNAs from multiple species including human, chimpanzee, rabbit, cow and mouse and comparing to the updated cat genome sequence (Genbank accession NM 000077.4) from the National Center for Biotechnology Information (NCBI) database. Feline p16 exon 3 was not included in the consensus sequence, because the genomic DNA sequence for exon 3 remains questionable because of disagreements in the alignment of exon 3 between species. Therefore, only sequences of exons 1α and 2 were used for primer design. Primers Fp16m2S (5’-GCCTGGGTCGGAGCCCGATT-3’) and Fp162AS (5’-TGCAGCACCACCAGCGTGTC-3’) were designed using NCBI Primer-Blast software to specifically detect a 166-nucleotide amplicon spanning the intron between exons E1α and E2 of the p16 gene. The amplicon was partially sequenced and included 95 nucleotides. The amplicon and the forward primer (total of 115 nucleotides) were aligned with p16 cDNA sequences from several species, which confirmed that this region of the predicted cDNA sequence and the junction of exons 1α and 2 were correct (Figure 1). Feline beta-2 microglobulin (forward primer: 5’-CTACTTCTGGCGCTGCTCTG-3’ and reverse primer: 5’-CCTGAACCTTTGGAGAATGC-3’) was use as a control housekeeping gene in this experiment.

### 2.4. Papillomavirus L1 Major Capsid DNA Investigation

The presence of papillomavirus L1 (major capsid) DNA was investigated in SCCF1, F2, and F3 cells as well as all FFPE FOSCC samples that had high p16 immunostaining. FBISC was used as a positive control for FcaPV2 DNA. Liver, spleen, kidney, lymph node, and lung tissues from a normal cat were used as negative controls. DNA was isolated using the DNeasy blood and tissue kit (Qiagen, Hilden, Germany) and quantified using a spectrophotometer (NanoVue, 4282 V2.0.0, Fisher Scientific, Pittsburgh, PA, USA). Four published primer pairs including JMPF/R [20] (pp. 259–263) (specific for L1-FcaPV2), MY09/11 [21] (pp. 3074–3083) (consensus primers of the L1 region in many PVs), and JMY2F/R [22] (pp. 360–366) and JMY3F/R [22] (pp. 360–366) (specific primers for L1-PV DNA originally amplified from a feline cutaneous SCC) were used to amplify papillomavirus L1 capsid DNA.

### 2.5. PCR Product Purification and Sequencing

PCR products were separated on 2% agarose gels and purified using a QIA quick gel extraction kit (Qiagen) and sequenced. Sequences were evaluated using the NCBI BLAST and compared to known sequences in the NCBI database.

### 2.6. Statistical Analysis

A one-way ANOVA with a Tukey’s post hoc analysis for multiple comparisons was used to examine the difference (1) between ages of cats with FOSCCs in each immunohistochemistry category and (2) p16 mRNA expression in SCCF1, F2, and F3 cells and normal cat gingiva. An unpaired t-test with Welch’s correction was used to compare p16 mRNA levels between SCCF3 cells and normal gingiva. For each IHC antibody, the percentage of cases within each scoring criterion and their 95% confidence intervals (CI) were calculated. No overlap between the CIs indicates a statistically significant difference between groups at a significance threshold of *p* = 0.05. Therefore, statistical significance was established at *p* < 0.05 for all comparisons.

## 3. Results

### 3.1. Relative p16 mRNA Quantification in 3 FOSCC Cell Lines

**Figure 2:**
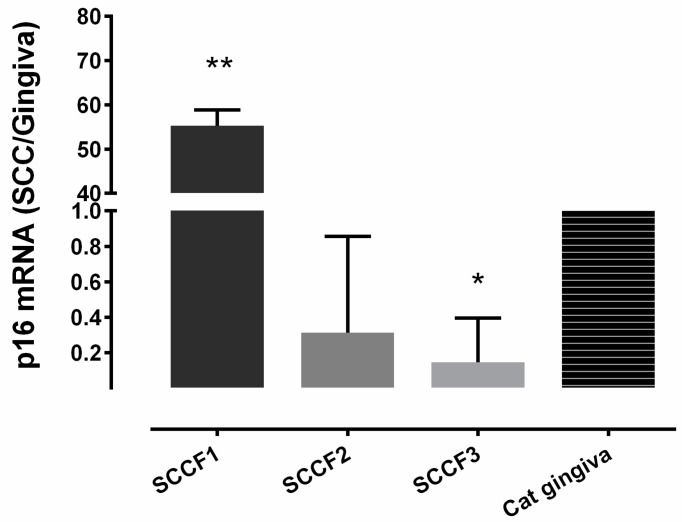
Relative p16 mRNA expression in 3 feline oral squamous cell carcinoma (FOSCC) cell lines compared to normal cat gingiva. All qRT-PCR was repeated in triplicate using 3 different passages of each cell line. The relative p16 mRNA expression in SCCF1 (*n* = 3) was significantly greater (55-fold) than in SCCF2 (*n* = 3), SCCF3 (*n* = 3), and normal gingiva (** *p* ≤ 0.0001). p16 mRNA was significantly reduced in the SCCF3 cells compared to normal gingiva (* *p* = 0.027).

Relative p16 mRNA expression in the SCCF1 cell line was significantly greater (55-fold) than the expression in normal cat gingiva, SCCF2, and SCCF3 (*p* < 0.0001) (Figure 2). P16 mRNA was reduced in the SCCF3 cells compared to normal gingiva (*p* = 0.027).

### 3.2. Immunohistochemistry (IHC)

**Figure 3:**
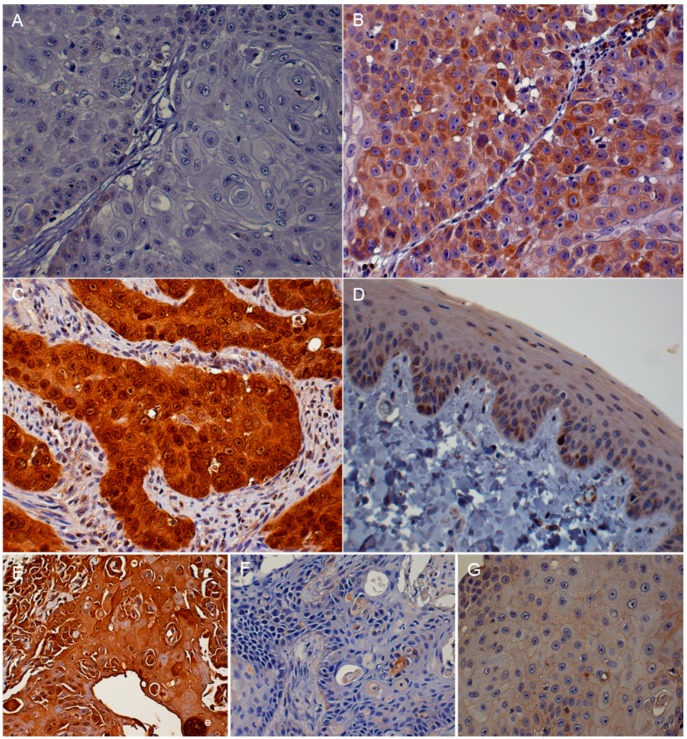
FOSCC, oral tissue, cat, immunohistochemistry photomicrographs. Absent to low p16 immunoreactivity. Very low intensity of p16 immunostaining was present in less than 20% of the neoplastic cells (**A**); moderate p16 staining. Moderate levels of p16 immunoreactivity were present in the cytoplasm of some of the FOSCC cells. Small numbers of nuclei had a high intensity of p16 immunostaining (**B**); high intensity p16 staining. Both nuclear and cytoplasmic p16 immunostaining were prominent in the tumor cells (**C**); normal gingival epithelium, cat. Immunoreactivity to p16 protein in normal cat oral tissues. There was a moderate intensity of p16 staining in normal gingival epithelium (**D**); p16 IHC in a mouse xenograft tumor derived from the SCCF1 cell line. High p16 intensity (brown color) was observed in both the nuclei and cytoplasm of SCCF1 tumor cells (**E**); p16 IHC in a mouse xenograft tumor derived from the SCCF2 cell line. Low to absent p16 intensity was present in SCCF2 cells (**F**); p16 IHC in a mouse xenograft tumor derived from the SCCF3 cell line. Low to absent p16 staining intensity was observed (**G**). (DAB/hematoxylin).

**Figure 4:**
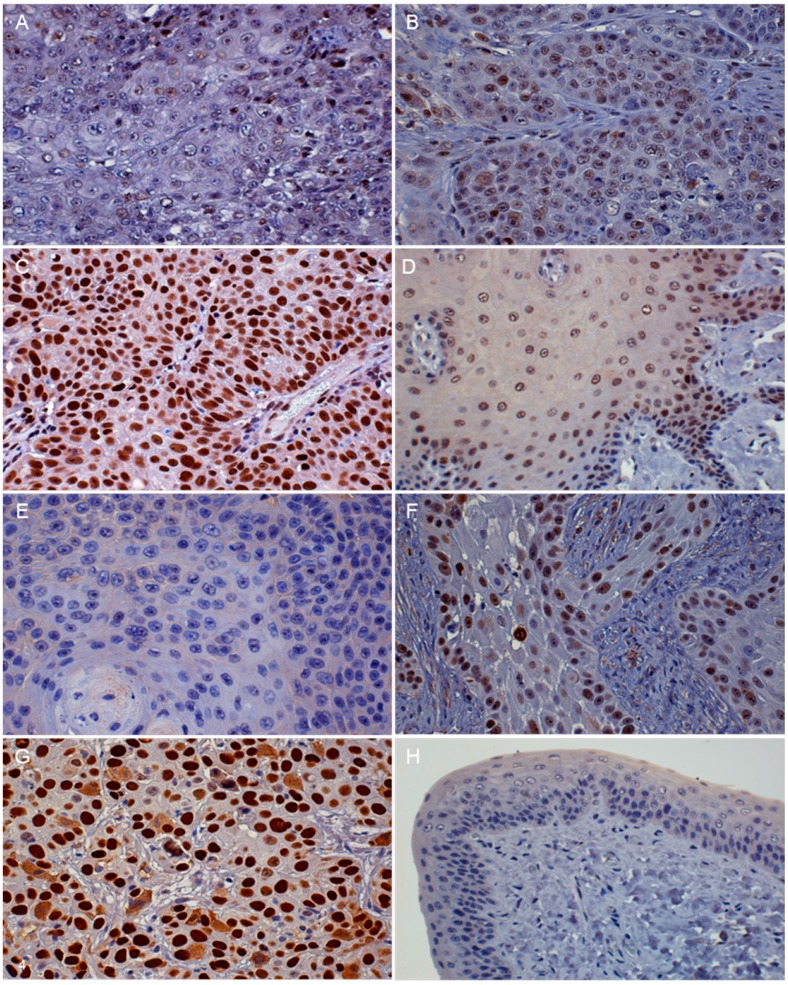
FOSCC, oral tissue, cat. FOSCC with absent to low intensity of pRb staining. Less than 50% of the neoplastic cells had a low intensity of nuclear immunostaining to pRb (**A**); microscopic features of moderate pRb intensity in FOSCC. Moderate pRb staining intensity was present in more than 50% of neoplastic nuclei (**B**); high intensity of pRb in FOSCC. Almost all of the neoplastic nuclei have high intensity immunostaining for pRb protein (**C**); normal gingival epithelium, cat. pRb IHC of normal cat oral tissue. Moderate pRb staining intensity was present in epithelial cell nuclei (**D**); microscopic features of absent to low intensity of p53 staining in FOSCC. The immunoreactivity to p53 protein was absent from neoplastic nuclei (**E**); microscopic features of moderate p53 staining intensity in FOSCC. Moderately intense immunoreactivity to p53 was present in most of the neoplastic nuclei (**F**); microscopic features of high intensity p53 immunostaining in FOSCC. Prominent p53 immunostaining (dark brown color) was visible within neoplastic nuclei (**G**). Normal gingival epithelium, cat. Immunostaining of p53 protein in normal cat oral tissue. Low intensity p53 staining was present (**H**) (DAB/hematoxylin).

**Figure 5:**
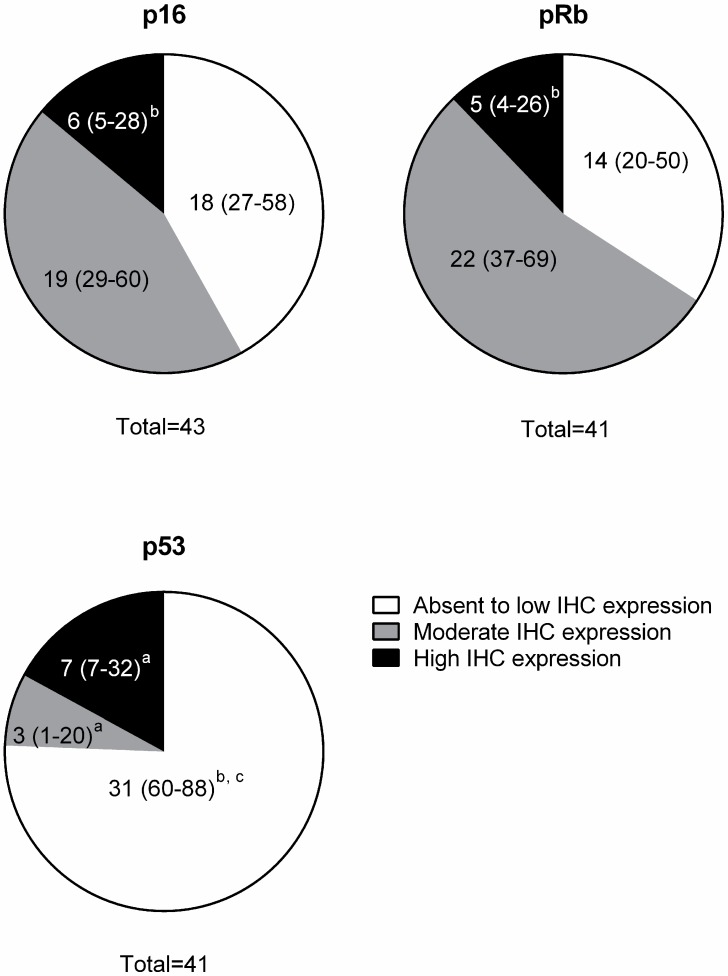
Immunohistochemistry of p16, p53, and pRb in spontaneous feline oral squamous cell carcinomas. The numbers in parentheses (percentages) indicate the 95% confidence intervals (CIs) of each group. No overlap between CIs indicates statistically significant differences between groups (*p* ˂ 0.05). Significantly different from absent to low group is indicated by (**a**) Significantly different from moderate group is indicated by (**b**) Significantly different from high group is indicated by (**c**).

Three IHC intensity groups were classified according to the percentage of positive cells and staining intensity as defined in Table 1. There was minimal to no staining variation in different regions of tumors for all antigens. Two samples evaluated for p16 expression were excluded from the pRb and p53 IHC due to insufficient tissue. The intensity of immunoreactivity was categorized as absent to low, moderate, or high (Figure 3A–C, Figure 4A–C,E–G ; Table 2). The p16 immunostaining was present in both the nucleus and cytoplasm of the tumor cells. It was found that 18/43 (42%) of the FOSCC had low p16 IHC expression. High p16 immunostaining was present in 6/43 (14%) of the FOSCC. This was significantly lower than the number of tumors with moderate p16 immunostaining (19/43, 44%) (Figure 5). Nuclear immunoreactivity was used to determine the presence of pRb. Fourteen of 41 (34%) FOSCCs had low to absent pRb staining intensity. In contrast, high intensity of pRb was found in 5/41 (12%) of samples. Most FOSCCs had moderate intensity of pRb staining (22/41, 54%; greater incidence, *p* < 0.05, compared to low or high intensity) (Figure 5). p53 IHC staining was also limited to the nuclei. High p53 staining intensity was present in 7/41 (17%) of the samples, while increased p53 immunostaining was not observed in 34/41 (83%) FOSCCs (Figure 5).

The histopathological characteristics of FOSCC in all of the different IHC groups were similar. The epithelium of normal cat gingiva and tongue had moderate staining intensity for p16 and a low to moderate intensity for pRb and p53 immunostaining (Figure 3D, Figure 4D,H). SCCF1 had a high intensity of p16 immunostaining in both the nucleus and cytoplasm of the neoplastic cells (Table 3). In contrast, p16 immunostaining was absent in both SCCF2 and SCCF3 cell lines (Figure 3E–G).

**Figure 6:**
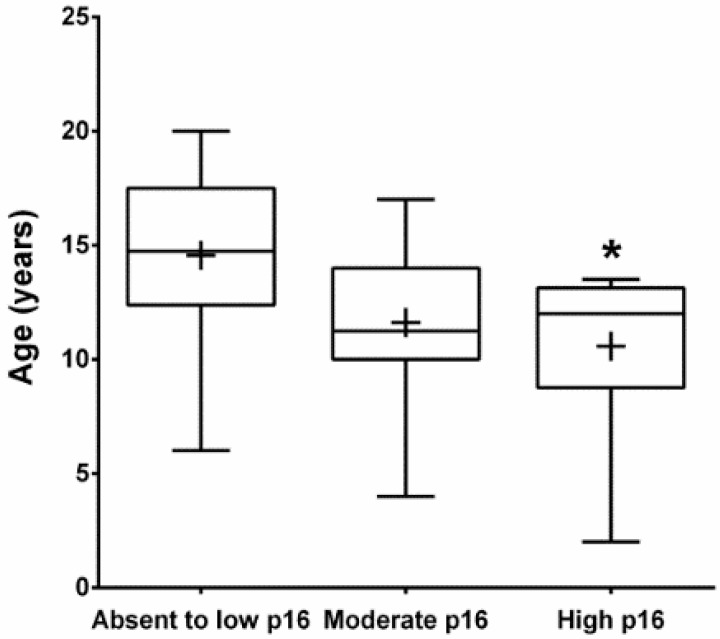
The average age of cats with FOSCC with high intensity p16 staining group (*n* = 6, mean = 10.6 years, SD = 4.0) was significantly lower than the age of cats in the absent to low p16 immunostaining intensity group (*n* = 18, mean = 14.5 years, SD = 4.3) (* *p* < 0.05). There was no significant difference between the average ages of cats with absent to low and moderate p16 (*n* = 18) and between moderate and high p16 staining intensity. The plus sign represents the means, the middle line of the boxes indicates the medians, the boxes contain 50% of samples, and top and bottom error bars represent maximum and minimum age of cats in each group, respectively.

The average age of FOSCC cats with high intensity p16 staining (mean = 10.6 years, SD = 4.0) was significantly lower than cats in the low intensity group (mean = 14.6 years, SD = 4.3), (*p* < 0.05) (Figure 6).

### 3.3. Papillomavirus L1 capsid DNA amplification:

PV-L1 capsid DNA was not amplified in the SCCF1, F2, or F3 cell lines or FOSCC samples with high p16 immunostaining intensity, but it was amplified in the FBISC positive control using the JMPF/R primers.

### 3.4. Sequencing

**Figure 7:**
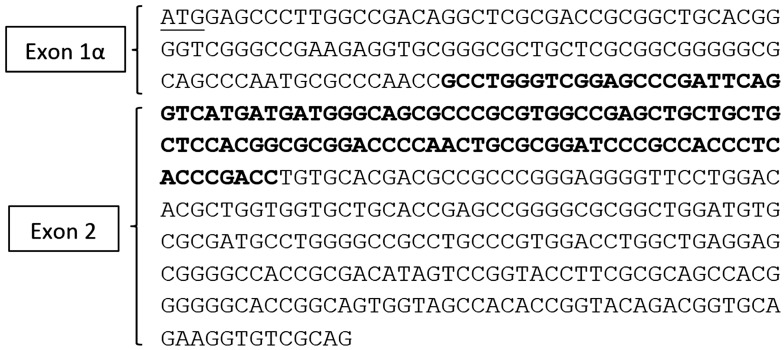
Feline p16 predicted cDNA sequence for exons 1α and 2. The feline p16 cDNA sequence shown was obtained from the updated cat genome sequence (Genbank accession NM 000077.4) after alignment with known cDNA sequences from other species. The sequenced feline p16 cDNA amplicon (Genbank accession: Banklt1884793 Seq1 KU508421) (115 nucleotides in bold) consists of the downstream end of exon 1α and the beginning of exon 2. The ATG start site of exon 1α is underlined.

A consensus p16 cDNA sequence was obtained by aligning p16 cDNAs from multiple species and comparing them to the updated cat genomic sequence (Genbank accession NM000077.4) from the NCBI database. Using The Blast Sequence Analysis Tool (http://www.ncbi.nlm.nih.gov/books/NBK21097/), a predicted feline p16 cDNA sequence was generated (Figure 7). RT-PCR products amplified in the SCCF1 cell line and normal cat gingiva were sequenced and aligned to the predicted feline p16 cDNA, which confirmed the predicted splice site for exons 1α and 2. The RT-PCR sequences had 100% homology with the predicted p16/CDKN2A cDNA of *Felis catus*. Although the full-length p16 cDNA could not be amplified due to the difficulty in designing functional RT-PCR primers for the cat and inadequate sequence information for the very short open reading frame of exon 3, a partial p16 cDNA sequence (Genbank accession: Banklt1884793 Seq1 KU508421) was amplified, confirmed, and found useful to measure p16 mRNA in feline cells and tissues (Figure 7).

## 4. Discussion

High intensity p16 and low intensity p53 and pRb immunostaining correlated with a subset of high-risk HPV-induced HNSCC in humans [10] (pp. 1931–1934). In contrast, low intensity p16 immunostaining was reported in non-viral-associated HNSCC, which had either mutation or inactivation of CDKN2A [11] (pp. 3630–3633), [23] (pp. 77–81). For this reason, p16 IHC serves as a useful tool for HNSCC classification and prognosis in humans.

The number of FOSCC with high intensity p16 staining found in this study (6/43, 14%) was greater than in a previous study (2/30, 7%) [18] (pp. 280–283). Based on HNSCC in humans, we hypothesized that the majority of FOSCCs would have either low or high p16. However, the results of the present study show that there were significant differences between cats and humans with oral cancer. Most FOSCC in cats had a moderate staining intensity of p16 and smaller subsets had high or low staining intensity. In humans, high intensity p16 staining was associated with high-risk HPV infection and a better prognosis, while loss of p16 immunostaining was associated with CDKN2A inactivation and a worse clinical outcome [6] (pp. 9–22), [7] (pp. 73–122). However, the connection between p16 immunostaining and prognosis in FOSCC has not been reported, therefore, the clinical outcome of FOSCC with different p16 staining intensity should be investigated further.

Interestingly, cats with FOSCC and abundant p16 protein were significantly younger than cats with FOSCC and low levels of p16. In humans, HPV was suspected to be involved in the pathogenesis of HNSCC in young patients [24] (pp. 249–258), [25] (pp. 1843–1849). Therefore, we speculate that the pathogenesis of FOSCC in younger cats with high p16 staining intensity may differ from FOSCC in older cats with low p16. It is also possible that FOSCC with high p16 intensity may be associated with a feline papillomavirus infection that has not been discovered yet. We were unable to amplify papillomavirus L1 capsid DNA from the SCCF1 cell line and FOSCC FFPE samples that had high intensity of p16 staining. The combined data from two studies showed that papillomavirus capsid DNA was only amplified in 3 of 47 FOSCC samples (p16 expression was not measured in these two studies) [26] (pp. 359–361), [27] (pp. 68–74). In another study, only two of thirty FOSCC samples had high p16 immunoreactivity, but FcaPV was not detected in these two specimens [18] (pp. 280–283). Therefore, papillomavirus infection may only be involved rarely in the pathogenesis of FOSCC. However, these relatively small sample numbers may be inadequate to draw a definitive conclusion on the role of papillomavirus in FOSCC. All primer pairs used to amplify papillomavirus DNA in these experiments were designed against the L1-regions of FcaPV2 and the conserved sequences from an alignment between different types of PVs. These primers may not detect other PVs that possibly exist in the FOSCC samples.

Low p53 and pRb were found in a subset of high risk-HPV-induced HNSCCs that had high p16 [10] (pp. 1931–1934). The interaction between the high-risk human papillomavirus E6 protein and p53 causes degradation of p53, while the interaction between E7 protein and pRb results in degradation of pRb and increases free p16 [14] (p. 2), [28] (pp. 405–415). A lack of pRb correlated with high p16 and FcaPV2 infection in feline cutaneous squamous cell carcinoma [15] (pp. 538–545). In this study, high p16 immunoreactivity was associated with low p53 in 5 out of 6 samples; however, only 1 of 6 FOSCC with high p16 had low pRb. Instead, FOSCC samples with high p16 were more likely to have moderate pRb (4 out of 6 samples). Therefore, the association between p16, p53, and pRb in FOSCC is unclear. Other proteins may be involved in up-regulation of p16 in FOSCC or the relationship between pRb and p16 in FOSCC may be different from HNSCC in humans.

We found that forty-two percent of FOSCC had low p16. These figures were greater than a previous study with 2/30 (7%) of cats having low p16 [18] (pp. 280–283). These differences may be due to regional differences and location of the FOSCC tissues within the oral cavity, as well as larger sample size, which might explain the greater proportion of low p16 that was found in the present study. The most common tumor location in the previous study was gingiva (12/30), while in this present study there was an even proportion between gingiva (9), tongue (11), sublingual (12) and mandible or maxilla (11). In addition, FOSCC in cats from different countries may have a distinct pathogenesis and protein expression. Loss of p16 immunostaining correlated with inactivation of the CDKN2A in HNSCC [11] (pp. 3630–3633). Hypermethylation of a CpG island in the CDKN2A promoter region and homozygous deletion mutation of p16 has been reported in various cancers, particularly HNSCC [6] (pp. 9–22), [11] (pp. 3630–3633), [29] (pp. 686–692), [30] (pp. 1197–1206). It is possible that many FOSCCs are associated with mutation or inactivation of the CDKN2A tumor suppressor gene. Unfortunately, there are still gaps in the feline whole genome sequence in the NCBI database and the genomic sequences surrounding the CDKN2A exons remain undefined. Completion of the feline p16 gene sequence will be essential for evaluating inactivation of the CDKN2A in FOSCC.

We found that p16 mRNA levels in SCCF1, SCCF2, and SCCF3 cell lines were consistent with p16 immunostaining in the xenograft samples. In particular, a high level of p16 mRNA expression was present in the SCCF1 cell line, which also showed a high p16 staining intensity.

Low p16 has been observed more often in the late stages of HNSCC (T3 or T4) [9] (pp. 58–60), which has a less favorable prognosis compared to HPV-induced-HNSCC [7] (pp. 73–122), [29] (pp. 686–692). p16 protein expression was suggested to possess prognostic utility in squamous cell carcinoma of the nasal planum in cats, since cats with high p16 had a longer survival compared to those with loss of p16 [31] (pp. 269–273). In addition, transfection of wild-type p16 into HNSCC cell lines decreased cancer cell proliferation in vitro [30] (pp. 1197–1206), [32] (pp. 3250–3253).

Increased p53 is the predictor of TP53 mutation in various cancers including HNSCC [33] (pp. 2455–2463). Seventeen percent (7/41) of FOSCC samples had a high intensity of p53 immunostaining. Therefore, somatic mutation of TP53 with increased expression and decreased activity of p53 may occur in some FOSCCs. Increased p53 immunostaining was not observed in 83% of FOSCCs. Lack of or low p53 immunostaining can be found in cells with TP53 inactivation as well as normal p53 expression. However, a subset of FOSCCs with low p53 immunostaining might be associated with TP53 inactivation, since 24%–53% of HNSCC in humans with low p53 immunostaining were related to TP53 mutations [33] (pp. 2455–2463), [34] (pp. 36). Twenty-seven percent (11/41) of FOSCC samples in this study had low p53 and p16 staining. Since CDKN2A encodes both the p16 and p14 proteins, inactivation of CDKN2A may also lead to loss of p14 if one of the common exons is inactivated or deleted. p14 stabilizes p53 by preventing the association between MDM2 and p53. Without p14, MDM2 independently binds and targets p53 protein for proteosomal degradation [35] (pp. 862–873). There is still no evidence that CDKN2A inactivation causes both p16 and p14 loss in HNSCC. However, the loss of both p16 and p14 IHC was found in 30% (9/30) of non-small cell lung cancers (NSCLC) and 67% (19/28) of pancreatic cancers [36] (pp. 1162–1168). Inactivation of CDKN2A is a potential mechanism to indirectly inhibit p53 protein expression. PV-related FOSCC was suspected in 12% (5/41) of samples that had low p53 and high p16 immunostaining intensity. However, papillomavirus capsid DNA was not amplified in these samples. Our data suggests that inactivation of the TP53 or CDKN2A may occur in FOSCC to directly or indirectly silence p53 expression. Inactivation of CDKN2A, as well as TP53, in the pathogenesis of FOSCC will require further investigation.

## 5. Conclusions

The immunohistochemical staining patterns for p16, Rb, and p53 have been described for FOSCC and provide important information on the potential pathogenesis and future improvements in the diagnosis, treatment, and prognosis of FOSCC. The immunohistochemistry patterns of p16, p53, and pRb in FOSCC were different from human HNSCC and feline cutaneous SCC. The p16 immunostaining intensity was consistent with relative p16 mRNA levels in three FOSCC cell lines. Partial sequence analysis of p16 DNA was completed and shown to be identical for a FOSCC with high p16 and normal feline gingiva. There is still limited evidence that papillomavirus plays a role in FOSCC, but it should not be excluded as a potential pathogenic mechanism. It is likely that subclassifications of oral SCC in cats based on tumor suppressor gene expression will guide research investigations and prognosis and treatment of cats with OSCC in the future.

## Figures and Tables

**Table 1:** Classification of p16, pRb, and p53 immunohistochemistry staining.

Protein	Absent to Low	Moderate	High
p16	<20% of cancer cells have low cytoplasmic intensity	20%–50% of cancer cells have low to intermediate cytoplasmic and nuclear staining intensity	>50% of cancer cells have high cytoplasmic and nuclear staining
pRb and p53	<20% of cancer cells have low to intermediate nuclear staining intensity	20%–50% of cancer cells have high nuclear staining intensity, or >50% of cancer cells have low to intermediate nuclear staining intensity	>50% of cancer cells have high nuclear staining intensity

**Table 2:** Signalment and immunohistochemistry data from 43 cats with FOSCC.

Signalment				IHC Results			
No.	Breed	Sex	Age (Year)	Location	p16	pRb	p53
1	DSH	FS	14	Gingiva	Low	Moderate	Low
2	DSL	MC	20	Gingiva	Low	Low	N/A
3	DSH	F	11	Gingiva	High	High	Low
4	Himalayan	FS	16	Gingiva	Moderate	Moderate	Low
5	DSH	MC	15	Gingiva	Moderate	Moderate	Moderate
6	DSH	FS	N/A	Gingiva	Moderate	Moderate	Low
7	Himalayan	MC	12	Gingiva	Low	High	Low
8	DSH	MC	11	Gingiva	Moderate	Moderate	High
9	DSH	MC	17	Gingiva	Moderate	Moderate	Low
10	DSH	MC	13	Tongue	Low	High	High
11	Siamese	FS	13	Tongue	Low	Low	Moderate
12	DSH	MC	16	Tongue	Low	Moderate	Low
13	DLH	MC	11	Tongue	Moderate	Low	Low
14	DSH	FS	13	Tongue	Low	Low	Low
15	Exotic SH	F	10	Tongue	Moderate	N/A	Low
16	DSH	MC	11.5	Tongue	Moderate	Moderate	Low
17	DLH	F	10	Tongue	Moderate	Moderate	Low
18	DSH	MC	6	Tongue	Low	Low	High
19	DSH	FS	15.5	Tongue	Low	Low	High
20	DSH	F	10	Tongue	Moderate	Moderate	Low
21	DSH	F	12	Sublingual	Low	Low	Moderate
22	Angora	FS	7	Sublingual	Low	Moderate	Low
23	DSH	MC	4	Sublingual	Moderate	Low	Low
24	DSH	M	14+	Sublingual	Moderate	Low	High
25	Persian	FS	13.5	Sublingual	High	Moderate	Low
26	DSH	MC	12	Sublingual	Moderate	Moderate	Low
27	DSH	M	12.5	Sublingual	Low	Low	Low
28	DLS	FS	17	Sublingual	Low	High	High
29	DSH	FS	19	Sublingual	Low	Moderate	Low
30	DSH	FS	14	Sublingual	Moderate	Moderate	Low
31	DLH	MC	17	Sublingual	Low	Moderate	Low
32	DSH	FS	19	Sublingual	Low	N/A	Low
33	DSH	MC	13	Mandi or Maxi	High	Moderate	High
34	DSH	MC	12	Mandi or Maxi	High	Low	Low
35	DLH	F	6.5	Mandi or Maxi	Moderate	Moderate	Low
36	DSH	MC	12	Mandi or Maxi	High	Moderate	Low
37	DSH	F	16	Mandi or Maxi	Low	Low	Low
38	DSH	F	20	Mandi or Maxi	Low	Low	Low
39	DSH	FS	2	Mandi or Maxi	High	Moderate	Low
40	DLH	MC	14	Mandi or Maxi	Moderate	Low	Low
41	Himalayan	F	11	Mandi or Maxi	Moderate	Moderate	Low
42	DSH	MC	13	Mandi or Maxi	Moderate	Moderate	Low
43	DSH	FS	9	Mandi or Maxi	Moderate	High	N/A

DSH: Domestic short hair, DLH: Domestic long hair, F: Female, FS: Spayed female, M: Male, MC: Castrated male, N/A: Not applicable, Mandi or Maxi: Mandible or Maxilla.

**Table 3:** Immunoreactivity to p16, p53, and pRb in three FOSCC Xenograft tumors and primary Feline Bowenoid in situ cutaneous Squamous Carcinoma (FBISC).

Sample	p16 IHC	p53 IHC	pRB IHC
SCCF1	High	Low	Low
SCCF2	Low	Low	Low
SCCF3	Low	Moderate	Low
FBISC	High	Low	Low

## Abbreviations

The following abbreviations are used in this manuscript:

FOSCC	Feline oral squamous cell carcinoma
HPV	Human papillomavirus
PV	Papillomavirus
FcaPV	Feline catus papillomavirus
IHC	Immunohistochemistry
FFPE	Formalin-fixed, paraffin-embedded
PCR	Polymerase chain reaction
RT-PCR	Reverse transcriptase PCR
HNSCC	Head and neck squamous cell carcinoma
